# An 8 × 8 CMOS Optoelectronic Readout Array of Short-Range LiDAR Sensors

**DOI:** 10.3390/s24206686

**Published:** 2024-10-17

**Authors:** Yeojin Chon, Shinhae Choi, Jieun Joo, Sung-Min Park

**Affiliations:** 1 Division of Electronic & Semiconductor Engineering, Ewha Womans University, Seoul 03760, Republic of Korea; wjsdulws7@gmail.com (Y.C.); rora0414@ewhain.net (S.C.); wxop01@naver.com (J.J.); 2 Graduate Program in Smart Factory, Ewha Womans University, Seoul 03760, Republic of Korea

**Keywords:** APD, A2V, CMOS, LiDAR, optoelectronics, T2V

## Abstract

This paper presents an 8 × 8 channel optoelectronic readout array (ORA) realized in the TSMC 180 nm 1P6M RF CMOS process for the applications of short-range light detection and ranging (LiDAR) sensors. We propose several circuit techniques in this work, including an amplitude-to-voltage (A2V) converter that reduces the notorious walk errors by intensity compensation and a time-to-voltage (T2V) converter that acquires the linear slope of the output signals by exploiting a charging circuit, thus extending the input dynamic range significantly from 5 μA_pp_ to 1.1 mA_pp_, i.e., 46.8 dB. These results correspond to the maximum detection range of 8.2 m via the action of the A2V converter and the minimum detection range of 56 cm with the aid of the proposed T2V converter. Optical measurements utilizing an 850 nm laser diode confirm that the proposed 8 × 8 ORA with 64 on-chip avalanche photodiodes (APDs) can successfully recover the narrow 5 ns light pulses even at the shortest distance of 56 cm. Hence, this work provides a potential CMOS solution for low-cost, low-power, short-range LiDAR sensors.

## 1. Introduction

Light detection and ranging (LiDAR) sensors have been exploited in various applications, such as unmanned autonomous vehicles, navigation systems for robots, indoor mapping on mobile devices, remote sensing, and home monitoring LiDAR sensors for senile dementia patients residing in long-term care facilities [1,2,3,4]. In particular, home monitoring sensors for senile dementia patients are mandated to be very small and provide in-depth information in an emergency, thus alerting the situations promptly to supervisors. For these purposes, a small, low-power, low-cost light detection and ranging (LiDAR) sensor can be a potential solution not only because its final images are blurred, thus avoiding image violation, but also because it can precisely provide every necessary information of single elders including move, fall, breath rate, etc. In addition, it can provide a number of advantages over conventional RF sensors due to their robustness against large ambient RF interferences.

Typically, LiDAR sensors employ the pulsed time-of-flight (ToF) scheme so that optical pulses can be transmitted from a laser diode driver in a transmitter to distant targets located within a detectable range. Then, the reflected optical pulses from the targets are received by the LiDAR receiver, which can be implemented as either a single-channel receiver or a multichannel array. By utilizing this principle, the distance to targets, also known as the detection range, can be estimated by measuring the time interval between the emitted (also known as START) pulses and the reflected (also known as STOP) pulses. Especially for the applications of indoor LiDAR sensors, the detection range would be as short as 0.2~5 m. Hence, the dynamic range of the LiDAR receiver is mandated to be wide enough (e.g., 1:625 [5]).

Multiple research projects have been conducted to suggest various topologies of analog front-end optical receivers [6,7,8,9,10,11,12,13,14,15]. However, most optical receivers employ off-chip avalanche photodiodes (APDs) that are integrated into PC boards via bond wires. This bond wire interconnection can be problematic because it may lead to considerable signal distortions and an increase in packaging costs in the cases of multichannel receiver arrays. In addition, the off-chip APD mandates electrostatic discharge protection diodes, which may deteriorate the bandwidth and noise performance.

Despite recent advancements in photodetection technologies, they have focused on utilizing wide-bandgap oxide semiconductors for ultraviolet communications and SnO₂/perovskite heterojunctions to improve sensitivity and response speed, particularly in high-performance applications [16,17]. While these types of research have been ongoing, we focus on a different aspect in this work, specifically on short-range detection where low power consumption and cost-effectiveness become crucial. For this purpose, we prefer employing on-chip APD to meet the desired requirements of low-cost and low-form-factor characteristics, hence making this work suitable for the applications of short-range LiDAR sensors.

This paper is organized as follows. Section 2 describes the circuit operations of the proposed optoelectronic readout array (ORA) along with the on-chip P^+^/N-well APD. Section 2 also presents the chip layout and the post-layout simulation results of the ORA. Section 3 demonstrates the measured results of the fabricated chips. Then, a conclusion is followed in Section 4.

## 2. Circuit Description

**Figure 1:**
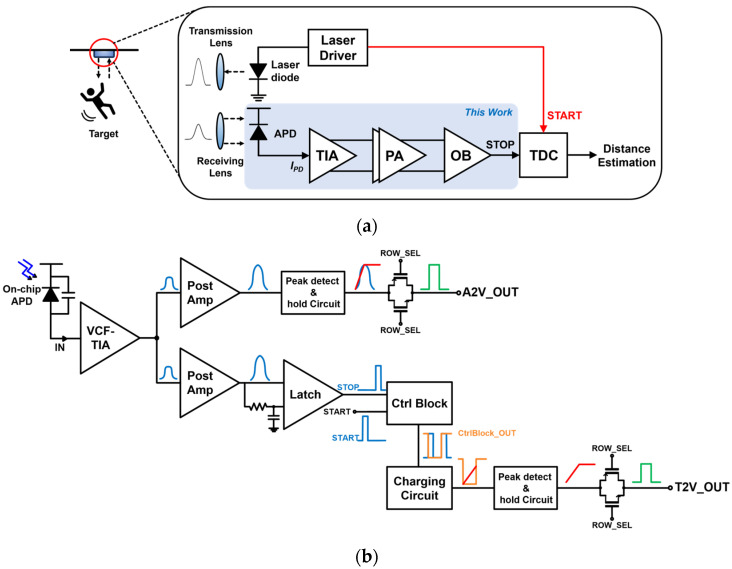
(**a**) Block diagram of a typical LiDAR sensor, (**b**) a single cell of the proposed ORA.

Figure 1a shows the block diagram of a typical LiDAR sensor system, in which a laser diode driver emits optical pulses to a target that is a senior in this figure, and the reflected optical pulses are received by the APD at the front end of the LiDAR receiver. Then, the generated photocurrents from the APD are converted to voltage pulses by the transimpedance amplifier (TIA). After being boosted further by the post-amplifier (PA), the voltage pulses enter the TDC, which can estimate the distance to targets by measuring the time interval between the emitted pulses and the reflected ones [18,19,20].

Especially, an 8 × 8 channel ORA is presented in this work for indoor monitoring LiDAR sensor applications, of which a single cell comprises an on-chip P^+^/N-well APD, a voltage-mode CMOS feedforward transimpedance amplifier (VCF-TIA), an amplitude-to-voltage (A2V) converter, and a time-to-voltage (T2V) converter. Figure 1b depicts the block diagram of the single cell in the proposed 8 × 8 CMOS ORA, where the A2V converter consists of a post-amplifier (PA), a peak detect and hold (PDH) circuit, and a transmission gate (TG) for selection, while the T2V converter comprises a PA, a latch, a control block, a charging circuit, a PDH, and a TG, respectively.

In particular, the A2V converter is suggested to process and recover the small input photocurrents (from 5 to 500 μA_pp_ at maximum) to output voltages linearly. After that, it holds the peak values until the reset signal enters. This A2V converter can be effective in detecting weak signals reflected from the target located within 8.2 m. However, it cannot recover the signals larger than 500 μA_pp_, which corresponds to 82 centimeters. In order to extend this limited dynamic range, the T2V converter is newly proposed for large input currents so that it can decode the time intervals between the START and STOP signals to output voltages and then hold the peak values. The proposed T2V converter can recover the relatively large input currents from 50 μA_pp_ to 1.1 mA_pp_, which corresponds to the minimum detection range of 56 centimeters. In short, the proposed ORA can successfully recover optical pulses reflected from the targets located within the detection range of 0.56~8.2 m.

### 2.1. On-Chip APD

**Figure 2:**
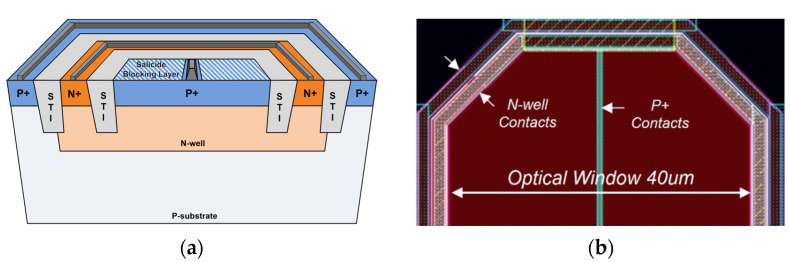
(**a**) A cross-sectional view of a P^+^/N-well APD, (**b**) the layout of the on-chip APD.

Figure 2 depicts the cross-sectional view of the on-chip P^+^/N-well (NW) CMOS APDs and its layout, in which the P^+^/N-well junction structure is exploited due to its several advantages such as a shallow trench isolation (STI) as a guard ring to enhance avalanche gain and a narrow depletion region in the NW to increase the bandwidth [15]. The P^+^ node is connected to the TIA input to convey the current pulses, whereas the P substrate is connected to the AC ground to prevent slow diffusion currents. A high voltage is applied to the NW to obtain the reverse bias of the P^+^/NW junction.

As depicted in Figure 2b, an octagonal shape of the on-chip P^+^/NW APD is preferred to avoid the edge-breakdown phenomenon. Its optical window (with a 40-μm diagonal line) is created using a salicide-blocking layer. Also, the silicide layer is applied to the P^+^ node to reduce the parasitic resistance.

As reported in our prior works, the fabricated on-chip P+/NW APD in the TSMC 180 nm 1P6M RF CMOS process exhibits a parasitic capacitance of 0.49 pF and a responsivity of 2.72 A/W at a bias voltage of 11.05 V with an avalanche breakdown voltage of 11.1 V [13,21].

### 2.2. VCF-TIA

**Figure 3:**
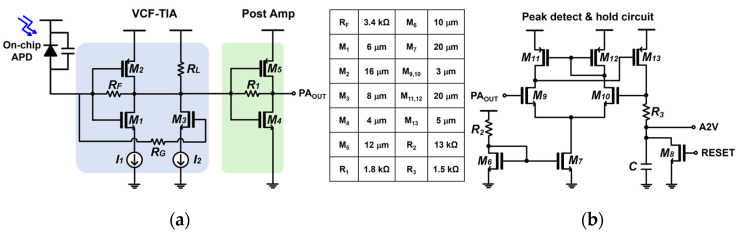
Schematic diagrams of (**a**) VCF-TIA with PA and (**b**) PDH.

Figure 3a illustrates the schematic diagram of the VCF-TIA followed by an inverter-based post-amplifier (PA). The VCF-TIA employs a conventional voltage mode inverter (INV) input stage, which includes a feedback resistor (R_F_) and a feedforward common-source (CS) amplifier, as described in Hong et al. (2018) [8]. The gate of this CS amplifier is connected to the gates of the INV stage, where R_G_ is the series resistor that is inserted not only because it can determine the bandwidth to meet the desired specification for the recovery of narrow laser pulses (i.e., 5 ns), but also because it can provide damping to the input parasitic inductance, if necessary, thereby preventing oscillation.

In addition, R_L_ is selected to be in the range of several tens of kiloohms, ensuring that a large bias current (I_M2_) flows through M_2_. This enhances the transconductance (g_m2_) of M_2_ because the drain DC voltage of M_2_ is fixed by the mechanism of the INV amplifier with a feedback resistor. Consequently, the increased transconductance (g_m2_) lowers the input resistance of the VCF-TIA and also helps to reduce the noise contributions from M_2_. Table 1 lists the DC operating points of the VCF-TIA and the PA, respectively.

**Figure 4:**
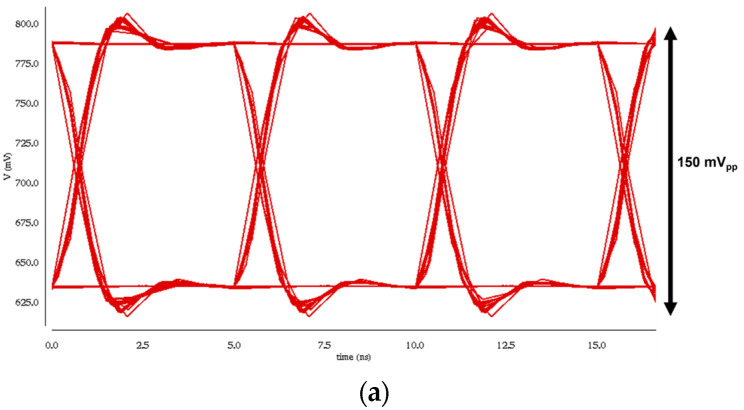

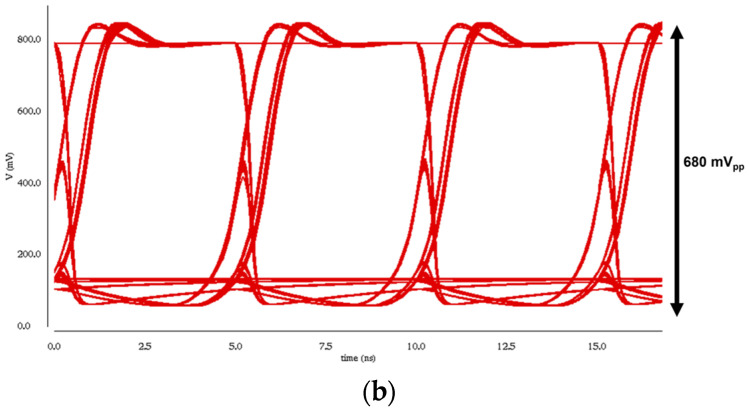
Simulated eye diagrams of the VCF-TIA at 200 Mb/s for two input currents of (**a**) 100 μA_pp_ and (**b**) 1 mA_pp_, respectively.

Figure 4 presents the simulated eye diagrams of the VCF-TIA at 200 Mb/s data rate for two different input currents of 100 μA_pp_ and 1 mA_pp_, respectively. These results confirm that the VCF-TIA can recover the eyes even with the large input currents.

### 2.3. A2V (Amplitude-to-Voltage) Converter

The primary objective of the proposed A2V converter is to facilitate the detection of targets located at a rather longer distance in the applications of short-range LiDAR sensors. As aforementioned, the A2V converter consists of a VCF-TIA, a post-amplifier (PA), and a peak detect and hold (PDH) circuit. Here, the gain of the PA is designed to be approximately 10 dB, thus providing a threefold increase in the signal amplitude. It should be noted that the gain setting in each block is very critical to maintain signal linearity and, hence, to ensure precise distance estimation based on these signal amplitudes.

Meanwhile, Figure 3b depicts the schematic diagram of the PDH circuit, which basically shares the topology of a two-stage operational amplifier except that the load is a series combination of a resistor (R_3_) and a capacitor (C) for the purpose of peak detection. Consequently, the PDH circuit can generate the final output pulses that correspond to the amplitudes of the incoming photocurrents.

**Figure 5:**
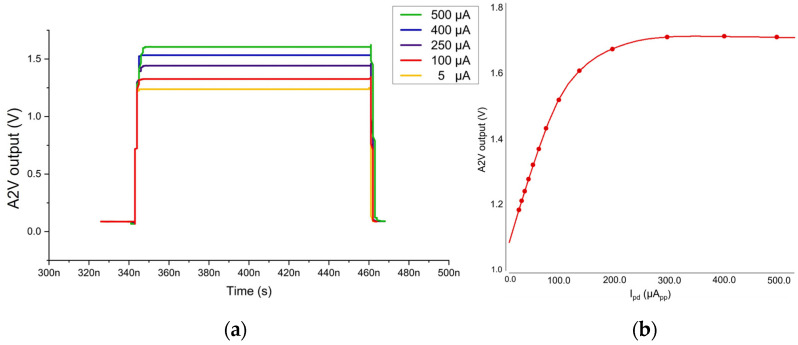

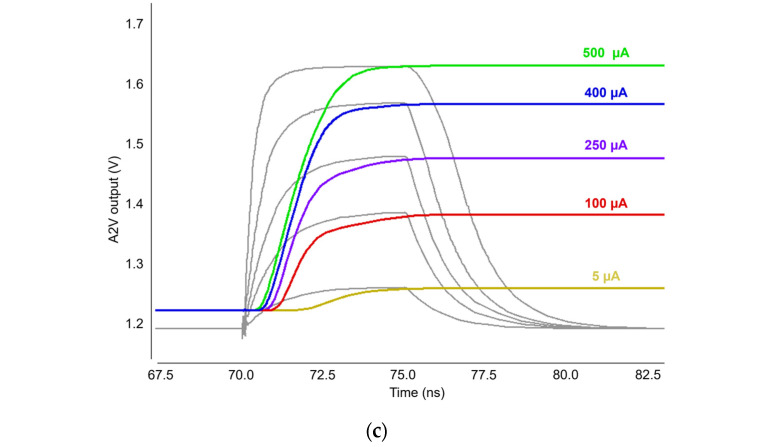
(**a**) Simulated pulse response of the A2V converter, (**b**) I–V curve and (**c**) transient response with the variations of the APD photocurrents.

Figure 5a shows the post-layout simulation results of the A2V path utilizing ‘Spectre Cadence’, which validates the anticipated capability of the A2V converter for the input photocurrents from 5 μA_pp_ to 500 μA_pp_ with an almost linear step with the aid of gain control scheme, maintaining the signal integrity and providing the precise distance measurements.

Figure 5b illustrates the I-V curve of the A2V converter, where it is clearly seen that the almost linear relationship exists for the input photocurrents up to 200 μA_pp_. The A2V output voltages are saturated with the input currents beyond 300 μA_pp_.

Figure 5c depicts the simulated transient response of the A2V converter with the variations of the APD photocurrents ranging from 5 μA_pp_ to 500 μA_pp_. In this case, the output voltages from the VCF-TIA pass through the PA of which output pulses (PA_out_) are shown in gray lines, and then through the PDH (represented in different colors).

### 2.4. T2V (Time-to-Voltage) Converter

The proposed T2V converter is a crucial component in extending the input dynamic range because it is responsible for converting the time-of-flight information into the corresponding output pulses. Namely, the photocurrents generated from the on-chip APD are converted to voltage signals by the VCF-TIA. Then, these voltage signals enter the PA for further gain boosting. Unlike the A2V path, the T2V converter requires the detection of signal presence. Therefore, the PA in the T2V path would only have to ensure that the output signals could be reliably processed by the subsequent stages, irrespective of reaching saturation. The output signals from the PA enter the latch, which results in digital pulses. In other words, the latch yields ‘1’ when a signal is present, whereas ‘0’ with no signals. Then, the latch output is fed into the control block together with a START signal from the Tx. The control block provides ‘0’ outputs for the duration between the START pulse and the received STOP pulse. Specifically, the output of the control block is switched to ‘0’ as soon as it receives the START pulse and then remains at GND until the latch output pulse is detected. Upon the detection of the latch output, the control block switches the output back to ‘1’, i.e., VDD. For circuit simulations, arbitrary signals were utilized to emulate the Tx START pulses.

**Figure 6:**
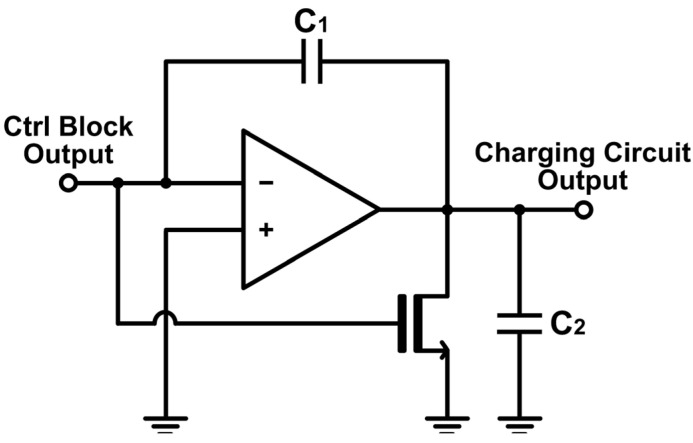
Schematic diagram of the charging circuit in the T2V converter.

Figure 6 illustrates the schematic diagram of the charging circuit in the T2V converter, which employs an operational amplifier with capacitor feedback. When the output of the control block remains ‘0’, the charging circuit increases its output voltage linearly. As soon as the control block generates ‘1’, the output of the charging circuit decreases, thus forming a triangular waveform. Consequently, the longer the time interval is taken to travel from Tx to Rx, the longer the charging circuit charges the output voltage. By correlating the time-of-flight directly with the output voltage, this T2V method enables precise and predictable range detection. Once the control block switches its output back to ‘1’, which indicates the arrival of the reflected signal at the Rx, the charging circuit stops the voltage increment and instead maintains the peak value. Then, the signal is held in the PDH circuit to preserve the peak voltage during the charging period. Finally, the peak voltage from the PDH circuit passes through a transmission gate (TG) that serves as the final stage of the T2V path. Hence, the proposed T2V circuit can convert the time-of-flight information efficiently into a proportional voltage signal. Each block plays a critical role in ensuring the precision and reliability of this conversion for range detection.

**Figure 7:**
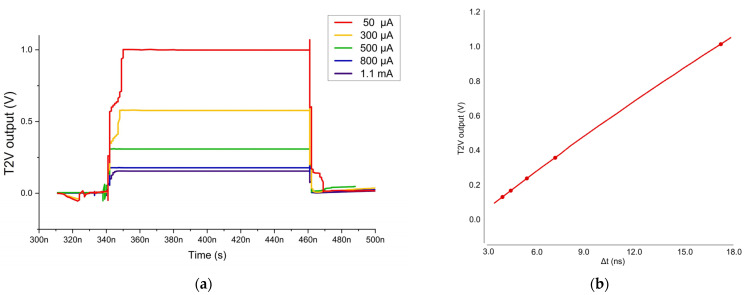
(**a**) Simulated pulse response and (**b**) a relationship curve of the T2V output voltage versus the time interval between the START and STOP pulses.

Figure 7 shows the post-layout simulation results of the T2V path by utilizing ‘Spectre Cadence’, where the T2V converter operates for the input currents of 50 μA_pp_~1.1 mA_pp_. This indicates that the minimum detection range of the proposed T2V converter reaches as short as 56 cm, while the maximum detection range reaches 8.2 m [22]. Hence, the ORA chip can acquire the input dynamic range of 27 dB with the aid of the proposed T2V converter. At this moment, it should be noted that there are several sources of errors in the proposed T2V converter. Among them, the main source would be ‘walk error’ because the T2V output varies depending on the timing of the reflected pulses from the target. Particularly, the latch block designed to convert the reflected pulses to large outputs may fail to capture the exact timing of the incoming signals if its slew rate decreases.

**Figure 8:**
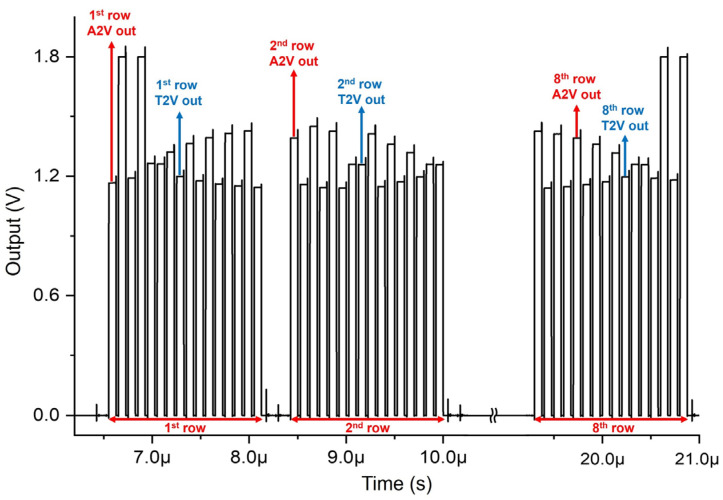
Simulated pulse responses of the proposed 8 × 8 CMOS ORA.

Figure 8 presents the simulated pulse response of the proposed 8 × 8 ORA, where the final outputs of three rows (1st, 2nd, and 8th rows) are selected as an example. In each row, the first column of the A2V and T2V outputs are generated, and then the rest column signals are consecutively followed. After that, a reset signal is generated after the 8th row for the next optical pulses. Hence, the A2V and T2V results are produced consecutively for 64 channels. Namely, the output of each channel is sequentially produced through a MUX. In these simulations, we have applied different input levels deliberately to the 64 channels so that the function of each channel can be verified.

## 3. Chip Fabrication and Measurements

**Figure 9:**
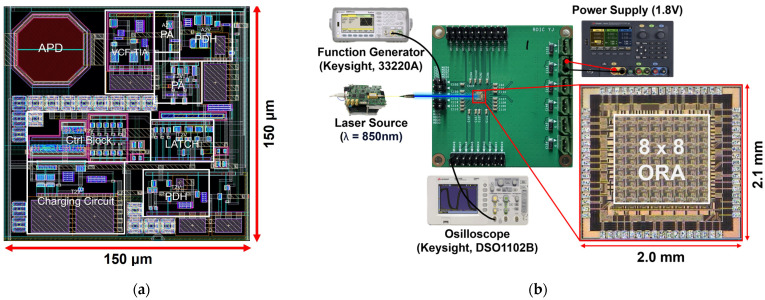
(**a**) Layout of a single-channel, and (**b**) test setup of the proposed 8 × 8 ORA chip.

Test chips of the proposed ORA were realized in the TSMC 180 nm 1P6M RF CMOS process. Figure 9a depicts the layout of the 8 × 8 ORA with on-chip P^+^/N-well APDs, where a single channel occupies a core area of 150 × 150 μm^2,^ and the whole chip occupies an area of 2.1 × 2.0 mm^2^, including I/O pads. Figure 9b shows the test setup for the proposed 8 × 8 ORA, where the output pulses were measured using an oscilloscope (DSO1102B, Keysight, Santa Rosa, CA, USA). The input signals were generated through a laser source driver (Seed LDD, Notice Ltd., Anyang-si, Korea) that was equipped with a laser diode (QPhotonics, Ann Arbor, MI, USA) operating at the wavelength of 850 nm. Optical pulse signals with a pulse width of 10 ns were generated.

**Figure 10:**
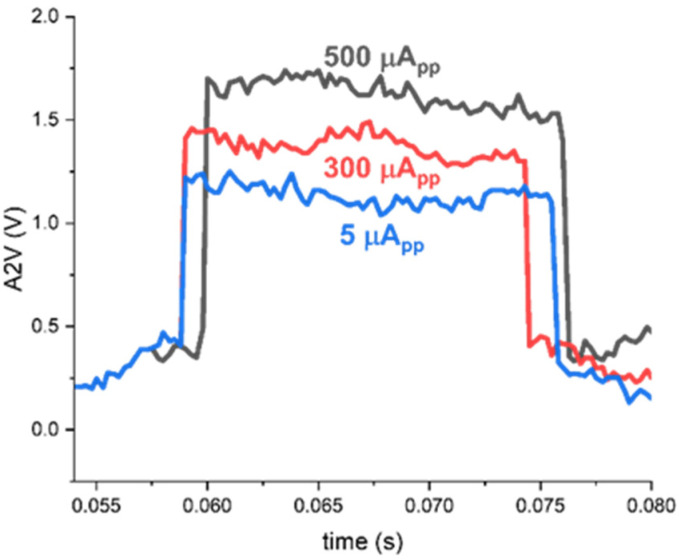
Measured A2V outputs of the 8 × 8 ORA for equivalent input currents from 5 μA_pp_ to 500 μA_pp_, respectively.

**Figure 11:**
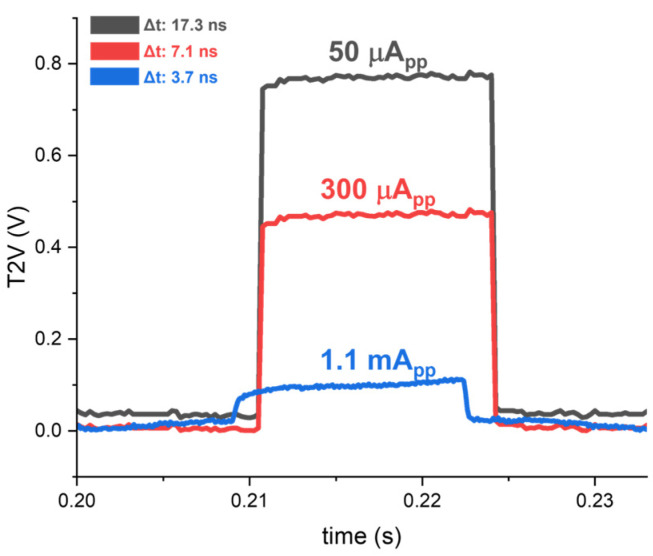
Measured T2V outputs of the 8 × 8 ORA for different 50 μA_pp_, 300 μA_pp,_ and 1.1 mA_pp_ input currents, respectively.

Figure 10 and Figure 11 demonstrate the measured results of the A2V and T2V paths in the 8 × 8 ORA chip for various input currents. First, Figure 10 demonstrates the measured outputs of the A2V path for three different input currents, which indicates an almost linear increase of the output voltages with respect to the input photocurrents, as anticipated in the simulations. Therefore, it can recover the weak input currents of 5~500 μA_pp_ that correspond to the minimum detection range of 82.5 cm and the maximum detection range of 8.2 m, respectively.

Second, Figure 11 reveals the measured output waveforms of the T2V path for three different input currents, where the highest peak is acquired for the smallest input current of 50 μA_pp_. This indicates that the proposed T2V converter enables the detection of a sudden movement (e.g., a fall accident of an elderly patient) at a distance of 2.6 m. For a large input current of 1.1 mA_pp_, the peak voltage of the output pulse becomes low, demonstrating the feasible detection at a much shorter distance.

Hence, the T2V path achieves a 26.8 dB input dynamic range with a linear voltage step of 255 mV for each 10 ns time interval, as shown in the simulations. These results correspond to the minimum detection range of 56 cm and the maximum detection range of 2.6 m.

**Figure 12:**
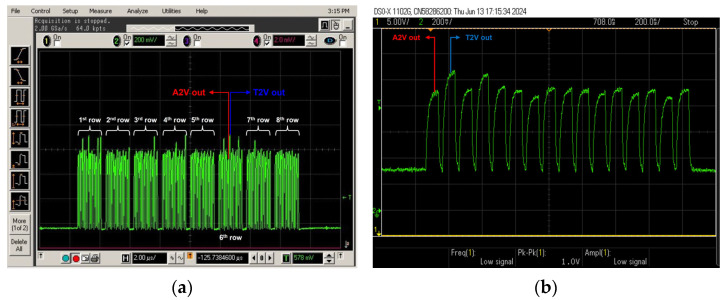
(**a**) Measured 8-row outputs of the 8 × 8 ORA at the optical testing, and (**b**) measured output of a single row (magnified).

Figure 12 shows the measured pulse response of the proposed 8 × 8 ORA. In Figure 12a, the final outputs of all the rows (from the 1st to the 8th rows) are demonstrated. The eight pulses of the A2V outputs are followed by those of the T2V outputs in each row. Figure 12b presents the magnified picture of a single row, where each A2V and T2V signal is clearly measured.

Conclusively, the proposed A2V and T2V converters operate complementarily to ensure the generation of stable output pulses, where the A2V converter extracts the intensity (i.e., amplitude) information of the signals, while the T2V converter captures the pulse width information that corresponds to the measured distance to the targets.

Table 2 summarizes the performance of the proposed ORA and compares it with prior research. Wang et al. (2020) [16] introduced a frequency-compensated voltage-mode inverter TIA that could successfully demonstrate low noise current spectral density. However, it consumed a large power and also mandated a reverse voltage of 200 V to bias the off-chip APD, thus resulting in a responsivity of 50 A/W. Also, the maximum detectable input current was obtained by saturating the combination of TIA and PA. Unfortunately, no output waveforms were demonstrated for the large input currents. Cho et al. (2014) [23] suggested a capacitive feedback TIA equipped with a dual-gain mode for a gain control mechanism. It successfully reduced the input-referred noise current with a small bandwidth and extended the dynamic range by utilizing the constant-delay detection method. However, it consumed a large chip area and a high power. Also, it required an on-chip high-pass filter to remove low-frequency noises and an off-chip InGaAs APD operating at 1550 nm with a high responsivity of 5 A/W. Ref. [24] presented a resistive feedback TIA with a replica circuit followed by a cascaded variable gain amplifier for gain control, where the bandwidth was considerably reduced to achieve low noise. Also, it exploited a timing discriminator with differential voltage shifters to compensate for walk errors. However, it demanded a large power dissipation. Zheng et al. (2018) [25] exploited a differential capacitive-feedback TIA with a very narrow 15-MHz bandwidth followed by a differential post-amplifier with offset cancellation, hence providing low noise characteristics despite the low responsivity of the APD. Yet, this receiver occupied a large chip area and was placed on a PC board with a separate TDC chip with two external comparators to achieve a wide dynamic range. Besides, the maximum detectable current was limited to 680 μA_pp_ only. Kurtti et al. (2020) [26] realized a differential voltage-mode TIA that successfully achieved a high transimpedance gain and a very large detectable input current. However, it exploited an off-chip APD with a high responsivity of 40 A/W. Also, AC coupling capacitors were required. In addition, it mandated bias resistors between the off-chip APD and the receiver IC. Furthermore, a separate TDC chip was necessary to acquire the maximum input dynamic range. Ma et al. (2020) [27] suggested a common gate current mirror TIA with off-chip gain control to increase the linear accuracy, which is then followed by a high-speed peak detector sample and hold circuit to improve the accuracy further. However, it mandated a programming FPGA-based gain control to extend the dynamic range characteristic and the linear accuracy. On the contrary, this work shows competent performance despite the low responsivity of the on-chip CMOS APD and the low transimpedance gain characteristics. In particular, it achieves a wide dynamic range even without the need for an automatic gain control scheme by the action of the T2V converter. Also, it provides very low power dissipation and small chip area characteristics, therefore enabling the feasible realization of a low-cost, low-power LiDAR sensor.

## 4. Conclusions

We have realized an 8 × 8 ORA chip implemented using a 180 nm CMOS technology. An on-chip CMOS P^+^/N-well APD is integrated into each cell, enabling the avoidance of unwanted signal distortion from bond wires and ESD protection diodes. Also, we have suggested several circuit techniques, including the A2V converter to reduce the notorious walk errors by intensity compensation and the T2V converter to acquire the linear slope of the output signals by exploiting a charging circuit, hence extending the input dynamic range characteristics significantly from 5 μA_pp_ to 1.1 mA_pp_, i.e., 48.6 dB. This dynamic range corresponds to the maximum detection range of 8.2 m by the action of the A2V converter and the minimum detection range of 56 cm with the aid of the proposed T2V converter. Optically measured results utilizing an 850 nm laser diode confirm that the proposed ORA chip successfully recovers narrow 5 ns light pulses even at the short distance of 56 cm. Hence, this work provides a potential solution for low-cost, low-power, short-range LiDAR sensors.

## Figures and Tables

**Table 1:** Operating points of the VCF-TIA and PA.

Operating Points	M1, M2, M3	M4, M5
Gate voltage (V)	0.83	0.82
Drain voltage (V)	0.82	1.25
Bias current (μA)	475 (I_1_), 560 (I_2_)	-

**Table 2:** Performance comparison with the recently reported LiDAR sensors.

Parameters		[16]	[23]	[24]	[25]	[26]	[27]	This Work
CMOS technology (nm)		180	350	180	350	350	180	180
APD	Type	Off-chip	Off-chip	Off-chip	Off-chip	Off-chip	Off-chip	On-chip
	C_pd_ (pF)	1.2	2.5~5	1	1.7	3	2	0.5
	Responsivity (A/W)	50 *	5	32	0.9	40	5	2.72
	Wavelength (nm)	905	1550	-	1550	905	905	850
Max. TZ gain (dBΩ)		81.6	124	106	131	100	100	63
Gain control		No	Yes	Yes	Yes	No	Yes ^‡^	No
Bandwidth (MHz)		281	160	150	15	230	110	842
Min. detectable current (µA_pp_)		2	0.053 @ SNR = 3.3	0.5 @ SNR = 5	0.019 @ SNR = 5	1.0 @ SNR = 10	1.0 @ SNR = 5	5
Max. detectable current (mA_pp_)		10 ^†^	0.64	1	0.68	39 ^ξ^	2	1.1
Dynamic Range (dB)		74	81.6	66	91	92	66	48.6
Power dissipation per channel (mW)		200	79	165	60	330	21	4.1
Chip area (mm^2^)		2.2 (1 ch.)	2.76 (1 ch.)	0.9 (1 ch.)	2.16 (1 ch.)	14 (Rx 1ch., TDC multi)	0.36 (1 ch.)	4.2 (64 ch.)

* with a 200-V reverse bias voltage, ^†^ with TIA+PA saturated (no transient waveforms shown for large input currents). ^ξ^ with a separate TDC chip, ^‡^ with FPGA-based gain control.

## Data Availability

Data are contained within the article.
